# Acute mitral valve regurgitation secondary to papillary muscle rupture due to infective endocarditis

**DOI:** 10.1186/s13019-022-01854-2

**Published:** 2022-07-08

**Authors:** Farshad Amirkhosravi, Qasim Al Abri, Alexander J. Lu, Lamees I. El Nihum, Renee K. Eng, Moritz C. Wyler von Ballmoos, Mahesh K. Ramchandani

**Affiliations:** 1 Department of General Surgery, Houston Methodist Hospital, Houston, TX USA; 2 DeBakey Heart and Vascular Center, Houston Methodist Hospital, 6550 Fannin St, Smith Tower Suite 1401, Houston, TX 77030 USA; 3 Texas A&M College of Medicine, Bryan, TX USA; 4 Department of Pathology and Genomic Medicine, Houston Methodist Hospital, Houston, TX USA

**Keywords:** Papillary muscle rupture, Infective endocarditis, Mitral regurgitation, Case report

## Abstract

**Background:**

Papillary muscle rupture due to infective endocarditis is a rare event and proper management of this condition has not been described in the literature. Our case aims to shed light on treatment strategies for these patients using the current guidelines.

**Case presentation:**

This case presents a 58-year-old male with acute heart failure secondary to papillary muscle rupture. He underwent an en bloc resection of his mitral valve with a bioprosthetic valve replacement. Specimen pathology later showed necrotic papillary muscle due to infective endocarditis. The patient was further treated with antibiotic therapy. He recovered well post-operatively and continued to do well after discharge.

**Conclusion:**

In patients who present with papillary muscle rupture secondary to infective endocarditis, clinical symptoms should drive the treatment strategy. Despite the etiology, early mitral valve surgery remains treatment of choice for patients who have papillary muscle rupture leading to acute heart failure. Culture-guided prolonged antibiotic treatment is vital in this category of patients, especially those who have a prosthetic valve implanted.

**Supplementary Information:**

The online version contains supplementary material available at 10.1186/s13019-022-01854-2.

## Introduction

The prognosis of infective endocarditis is generally poor. The incidence of infective endocarditis-related hospitalization has increased from 34,488 in 2003 to 54,405 in 2016 [1]. Risk factors associated with infective endocarditis include age over 65, intravenous drug use, structural heart disease, valvular disease, prosthetic valve repair, and previous history of infective endocarditis. In the United States, the mortality rate of infective endocarditis between 1980 and 2014 was 2.4 per 100,000 [2].

Endocarditis can cause structural heart damage. Direct leaflet involvement and damage is usually the cause of valve incompetency in these patients. Rarely, infective endocarditis can also involve valve-supporting structures, such as the papillary muscles. Here, we report an atypical cause of papillary muscle rupture. Due to the rarity of this complication, the management of papillary muscle rupture secondary to infective endocarditis can be challenging, complex, and predicated on clinical judgement.

## Case report

**Fig. 1:**
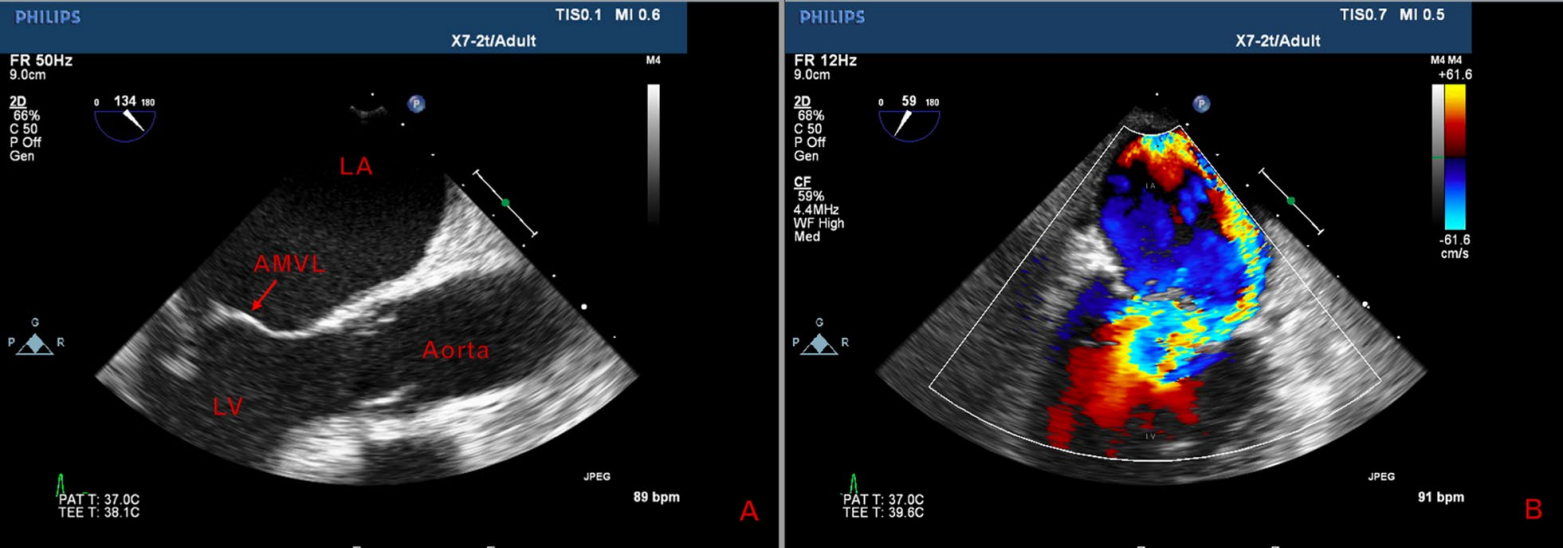
Mitral valve imaging. Flail anterior mitral valve leaflet is seen on transesophageal echocardiogram (**A**) with evidence of a posteriorly directed regurgitant jet (blue) on transthoracic echocardiogram (**B**)

A 58-year-old male with a past medical history significant for chronic obstructive pulmonary disease presented with shortness of breath exacerbated by exertion. Additional complaints included fever and productive cough. Vital signs were remarkable for tachycardia and oxygen saturation of 96% on two liters of nasal cannula. On physical examination the patient was in no apparent distress. Rales were heard on chest auscultation and cardiac exam was significant for a 3/6 systolic murmur best heard at the apex. Blood work was significant for elevated white blood cell count. Chest X-ray was consistent with pulmonary edema. Cardiac catheterization revealed non-obstructive coronary artery disease. Transthoracic echocardiogram (TTE) showed severe mitral valve regurgitation with a posteriorly directed eccentric jet, and follow-up transesophageal echocardiogram revealed a flail anterior mitral valve leaflet (AMVL) (Fig. 1).

The patient was admitted for medical optimization and planned surgical intervention due to acute heart failure secondary to severe mitral regurgitation. Blood cultures showed *Staphylococcus haemolyticus* growth. After adequate medical optimization, the patient was taken to the operating room for surgical management of the mitral valve.

**Fig. 2:**
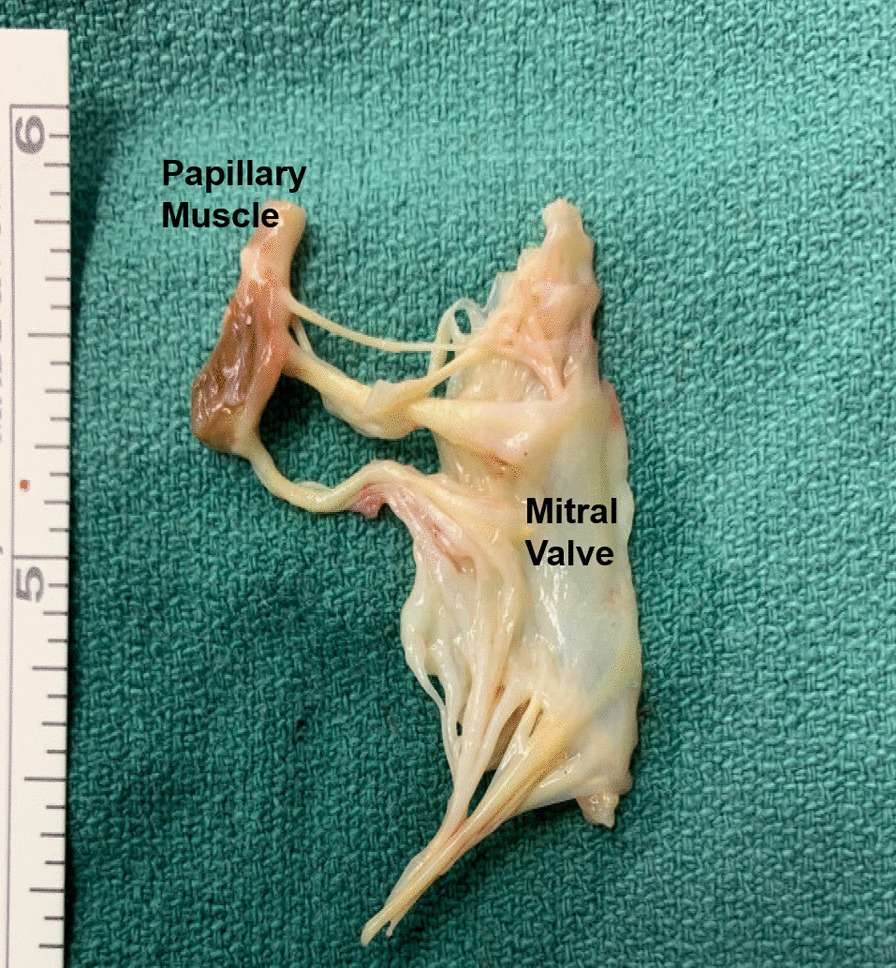
Resected papillary muscle and mitral valve

The operation was done via a mini right thoracotomy incision made through the fourth intercostal space and cardiopulmonary bypass was achieved via left femoral cannulation. Excellent exposure of the mitral valve was achieved through Sondergaard’s groove. Examination of the mitral valve showed maximal prolapse at the A3 region of the AMVL due to complete detachment of the corresponding papillary muscle. The mitral valve and papillary muscle were resected en bloc and sent for culture and pathology (Fig. 2). The mitral valve was replaced with a 31 mm St. Jude Medical Epic™ bioprosthetic valve (St. Jude Medical, Inc., MN, USA). The patient tolerated the procedure well without complications.

**Fig. 3:**
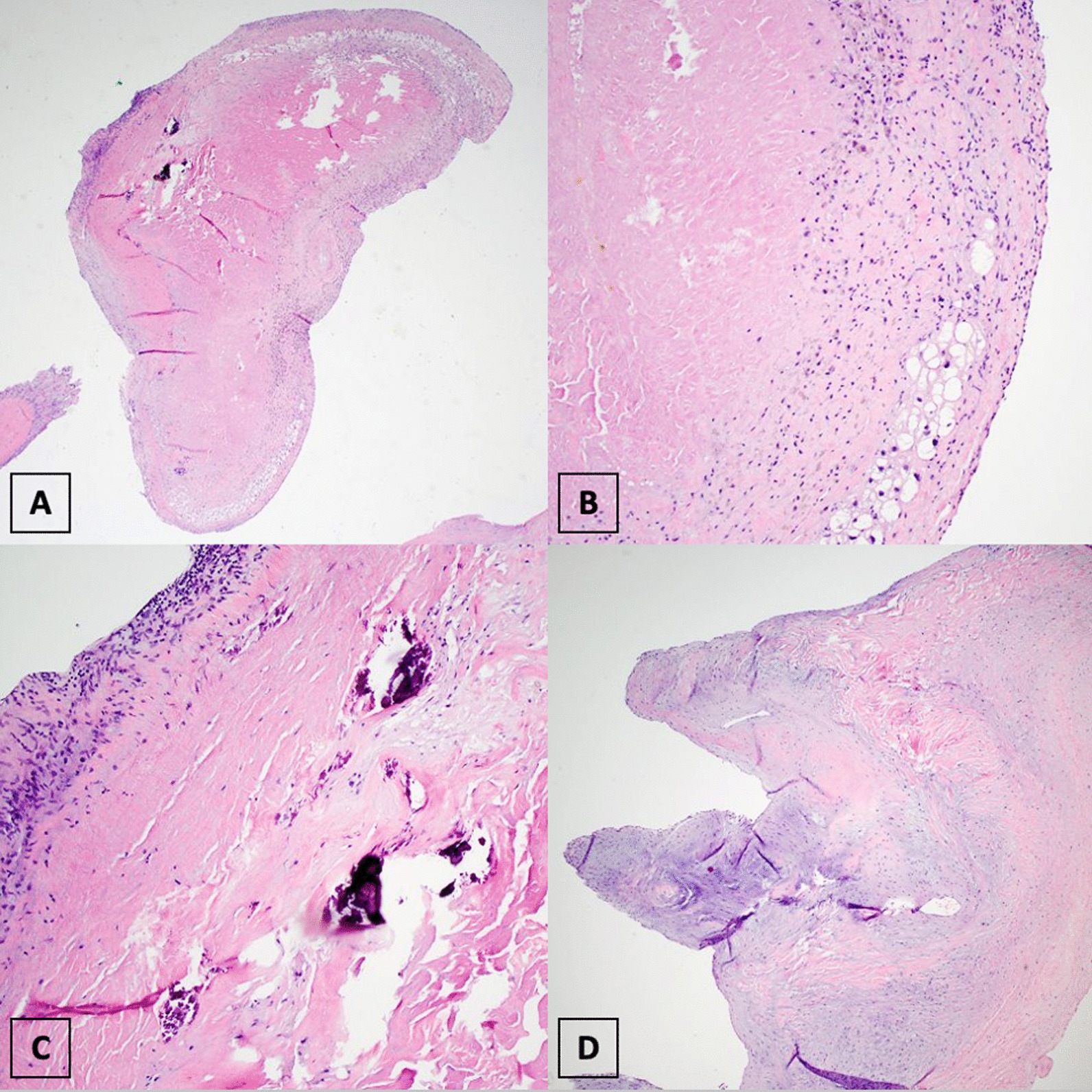
Histological evaluation of the papillary muscle. Histologic findings revealed myocardial necrosis with histiocytic inflammation (**A**, **B**), calcification (**C**), and valvular tissue with myxoid degeneration (**D**)

Culture of the surgical specimen showed *S. haemolyticus* growth consistent with blood culture drawn during admission. Surgical pathology showed extensive histological inflammation and myocardial necrosis consistent with infective endocarditis (Fig. 3). Infectious disease was consulted and a six-week course of intravenous vancomycin was started.

Postoperative course was unremarkable. Repeat cultures after the start of antibiotics therapy did not grow any microorganism. The patient was discharged home on postoperative day eight. At 6-week follow up, he denied any new symptoms of fever or chills. TTE showed a well-functioning bioprosthetic mitral valve and preserved ventricular function (Additional file 1: Table S1).

## Discussion

Papillary muscle rupture is classified into three categories: ischemic, non-ischemic, and iatrogenic. The non-ischemic subset involves patients with blunt chest trauma, myxomatous disease, spontaneous rupture, and rarely, infective endocarditis as in our patient. Papillary muscle rupture results in severe mitral regurgitation, regardless of the etiology. Unlike with ischemic causes of papillary rupture, patients with non-ischemic etiology have preserved ventricular function, and thus less burden of cardiogenic shock. Although the operative management of papillary muscle rupture may be similar, the etiology of disease differentiates the timing of surgical intervention and outcome.

The timing of sterilization with antibiotics and surgical intervention depends on symptomatic severity and stability [3]. In patients with acute left-sided valvular regurgitation due to infective endocarditis resulting in heart failure, early surgical intervention prior to antibiotic sterilization is the current guideline recommendation [4, 5]. In patients who receive early surgical intervention, risk of mortality is significantly lower compared to late surgical intervention [6].

Although reimplantation of the ruptured papillary muscle might be technically feasible, it is not viable in this patient population, as most are in cardiogenic shock at the time of operation. Furthermore, the rate of recurrent papillary muscle rupture is higher, especially with necrotic tissue. Importantly, in patients with papillary muscle rupture due to infective etiology, extensive debridement and removal of infective tissue is key to eradicating infection, and thus reimplantation is not a viable intervention.

Thus, these patients are better served with valve replacement. Bioprosthetic and mechanical valves have similar long-term outcomes and risk for endocarditis recurrence [7]. Thus, choice of valve, bioprosthetic or mechanical, is up to patient preference. Many patients prefer bioprostheic valves to avoid lifelong anticoagulation, as in our case.

Choice of antibiotic and duration of treatment is another critical factor in the management of patients with infective endocarditis. Blood or tissue cultures are essential in identifying the microorganism involved and directing therapy. In patients with coagulase-negative *Staphylococcus* infection, as in our patient, treatment with prosthetic valve replacement and a six-week course of vancomycin therapy is recommended [8]. Repeat blood cultures every 24 to 48 h are necessary to monitor response to antibiotic therapy.

## Conclusion

Papillary muscle rupture due to infective endocarditis is a rare clinical event; however, prompt treatment can improve outcome. Early surgical intervention in patients with signs of heart failure is imperative. Antibiotic treatment is also vital for successful therapy and prevention of recurrent endocarditis. Implementing both of these treatment strategies allowed our patient to have an excellent outcome.

## Supplementary Information

**Additional file 1. Table S1:** Timeline of the patient’s course of illness.

## Data Availability

Not applicable.

## Abbreviations

AMVL: Anterior mitral valve leaflet
TTE: Transthoracic echocardiogram
