# Supplementary material for: Acute mitral valve regurgitation secondary to papillary muscle rupture due to infective endocarditis
Source: J Cardiothorac Surg. 2022 Jul 8;17:173. doi: 10.1186/s13019-022-01854-2 (PMC9270734; doi:10.1186/s13019-022-01854-2)
Supplement: Supplementary file 1 — Additional file 1. Table S1: Timeline of the patient’s course of illness. [file 13019_2022_1854_MOESM1_ESM.docx]

*Timeline*

| Presentation | Shortness of breath, fever and productive cough |
| --- | --- |
|  | Chest X-ray: pulmonary edema |
|  | Cardiac catheterization: non-obstructive coronary artery disease |
|  | Transthoracic echocardiogram (TTE): severe mitral valve regurgitation with posteriorly directed eccentric jet |
|  | Transesophageal echocardiogram: flail anterior mitral valve leaflet (AMVL) |
|  | Patient admitted for medical optimization and planned surgical intervention for acute heart failure secondary to mitral regurgitation |
|  | Blood cultures: *Staphylococcus* *haemolyticus* |
| 1 week | Surgical exploration via right thoracotomy: AMVL prolapse due to complete detachment of the corresponding papillary muscle |
|  | Surgical intervention: en bloc resection of mitral valve and papillary muscle and replacement with a 31 mm St. Jude Medical Epic^TM^ bioprosthetic valve |
|  | Culture of surgical specimen: *Staphylococcus* *haemolyticus* growth consistent with blood culture |
|  | Infectious disease consult: six-week course of intravenous vancomycin initiated |
| 2 weeks | Patient discharged on post-operative day 8 |
| 6 weeks | Clinically well and asymptomatic with no symptoms of fever or chills |
|  | TTE: well-functioning bioprosthetic mitral valve and preserved ventricular function |
